# The effectiveness of rituximab and HIV on the survival of Ontario patients with diffuse large B‐cell lymphoma

**DOI:** 10.1002/cam4.3362

**Published:** 2020-08-13

**Authors:** Steven Habbous, Helen Guo, Jaclyn Beca, Wei Fang Dai, Wanrudee Isaranuwatchai, Matthew Cheung, Kelvin K. W. Chan

**Affiliations:** ^1^ Ontario Health (Cancer Care Ontario) Toronto ON Canada; ^2^ Centre for Excellence in Economic Analysis Research St. Michael's Hospital Toronto ON Canada; ^3^ Canadian Centre for Applied Research in Cancer Control Toronto ON Canada; ^4^ Institute of Health Policy, Management and Evaluation Toronto ON Canada; ^5^ Hematology Sunnybrook Health Sciences Centre Toronto ON Canada; ^6^ Medical Oncology Sunnybrook Health Sciences Centre Toronto ON Canada

**Keywords:** AIDS, CD4, CHOP, HIV, lymphoma, rituximab, survival

## Abstract

**Introduction:**

For patients with diffuse large B‐cell lymphoma (DLBCL), standard‐care is rituximab administered with CHOP or CHOP‐like chemotherapy (R‐CHOP). However, the effectiveness and safety of R‐CHOP among DLBCL patients with human immunodeficiency virus (HIV) infection is less clear, as HIV+ patients were omitted from most clinical trials and population‐level data from unselected patients are limited. R‐CHOP was funded for HIV‐associated DLBCL patients with CD4 >50/mm^3^ in Ontario in February 2015.

**Methods:**

Patients with a new diagnosis of DLBCL were identified from the Ontario Cancer Registry between April 2010 and March 2018. HIV diagnosis and chemotherapy regimen were ascertained using administrative databases at Ontario Health. The effect of rituximab and HIV on overall survival was assessed in the HIV+ subgroup (R‐CHOP vs CHOP) and in the R‐CHOP subgroup (HIV+ vs HIV−).

**Results:**

Among HIV+ patients, receipt of R‐CHOP was associated with a fivefold improvement in overall survival (hazard ratio [HR] 0.29 (0.13‐0.66) compared with CHOP), after adjustment. Among patients who received R‐CHOP (n = 6106), older age, male sex, lower neighborhood income, and higher comorbidity were associated with worse overall survival, after adjustment (*P* < .001 for all), but HIV positivity was not prognostic (HR 1.12 (0.60‐2.10)). Within 1‐year after diagnosis, HIV+ patients receiving R‐CHOP had a similar proportion of patients who visited the emergency department (67% vs 66% *P* = .43) or admitted to hospital (58% vs 52%, *P* = .43) and as HIV− patients receiving R‐CHOP.

**Conclusion:**

HIV status did not affect prognosis for patients with DLBCL receiving R‐CHOP in an unselected general population when rituximab was used according to funding criteria. R‐CHOP was safe and effective for DLBCL treatment, regardless of HIV status.

## Graphical abstract

The standard of care for patients with aggressive‐histology B‐cell lymphoma is rituximab in addition to a CHOP or CHOP‐like chemotherapy regimen. For HIV+ patients, there are less data available on the effectiveness of this treatment, but in 2015, policy changes in Ontario extended this treatment modality to this subgroup of patients. In the present study, we demonstrate that rituximab with CHOP is as safe and effective for HIV+ patients as it is for HIV− patients.

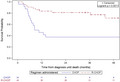

## 1 INTRODUCTION

In 2018, there were an estimated 37.9 million people living with human immunodeficiency virus (HIV) and 770 000 died due to HIV‐related illnesses worldwide.^1^ Since the introduction of highly active antiretroviral therapy (HAART) in 1996, there have been marked improvements in overall survival and reductions in morbidity among HIV+ patients.^2^, ^3^ This may be attributable to the effect of HAART on reducing the detectable viral load of HIV and elevating CD4+ T‐cell counts, which may in turn improve the ability to ward off opportunistic infection and reduce the oncogenic potential of viruses like HIV, human herpesvirus subtype‐8 and human papillomavirus.^4^, ^5^, ^6^ Although the incidence of acquired immunodeficiency syndrome (AIDS)‐related malignancies has declined since the introduction of HAART, the incidence of cancers among HIV+ persons exceeds what is expected amongst the general population, with lymphoma accounting for more than half of all HIV‐related malignancies.^7^, ^8^, ^9^, ^10^, ^11^, ^12^

Previous clinical studies demonstrated the efficacy of combination therapy using the anti‐CD20 antibody rituximab with standard chemotherapy regimens, and as a result, rituximab‐based therapies have become standard of care.^13^, ^14^ However, HIV‐related lymphoma is more aggressive than HIV‐unrelated lymphomas, and this particular group has often been excluded from lymphoma trials.^15^, ^16^ Existing studies on the effectiveness of rituximab in HIV+ patients were limited to small phase II trials, meta‐analyses of such trials, and one phase III study that produced equivocal findings at the time, demonstrating improved lymphoma control with rituximab but at the cost of higher infection‐related mortality.^17^, ^18^, ^19^, ^20^, ^21^ Other investigators used a mixed approach, comparing clinical trial cohorts with observational cohorts, deriving conflicting results on patient outcomes.^15^, ^22^ Thus, a comparative analysis is needed using unselected patients.

The purpose of this study was to evaluate the real‐world effectiveness and safety of rituximab for the treatment of HIV‐related lymphoma in an unselected population‐based retrospective study using administrative databases in Ontario, Canada. We explored the effect of a funding policy change that enabled rituximab administration to all lymphoma patients in the province with HIV as long as the CD4 count was >50/mm^3^. We also explored the effect of HIV on patient outcomes among patients receiving rituximab with standard chemotherapy.

## 2 METHODS

### 2.1 Cohort selection

We included all patients newly diagnosed with an aggressive‐histology lymphoma in the Ontario Cancer Registry between April 1, 2010 (earliest date we are able to capture HIV status due to data availability) and March 31, 2018, restricting only to malignant cases (ICD‐O‐3 behavior code = 3). We restricted the cohort to include only lymphoma subtypes that are potentially HIV‐related, including non‐Hodgkin lymphoma with the following ICD‐O‐3 morphology codes: diffuse large B‐cell lymphoma (DLBCL; 9680), non‐Hodgkin lymphoma not otherwise specified (9591), Burkitt lymphoma (9687), T‐cell‐rich large B‐cell lymphoma (9688), mediastinal (thymic) large B‐cell lymphoma (9679, associated with topography code C38.3), large B‐cell lymphoma arising in HHV8‐associated multicentric Castleman disease (9738), diffuse large B‐cell immunoblastic lymphoma (9684), and malignant lymphoma not otherwise specified (eg, not necessarily non‐Hodgkin lymphoma, 9590). We excluded histologies that do not express CD20, the target for rituximab, including plasmablastic lymphoma (9735) and primary effusion lymphoma (9678).^23^ Finally, polymorphic posttransplant lymphoproliferative disorder (9971) was also omitted because this disease is poorly understood and is extremely rare (eg, <6 HIV+ cases were observed). We only included adults (18+ years old) who were Ontario residents at the time of their initial diagnosis. For patients with multiple aggressive lymphoma diagnoses, we selected the first case.

### 2.2 HIV status

We used previously validated methodology to assign HIV status. Patients were HIV+ if there were three ICD‐9 diagnostic codes for HIV (042, 043, or 044) observed within 3 years in the Ontario Health Insurance Program database. Compared to chart review, this resulted in a sensitivity of 96.2% and specificity of 99.6%.^24^ The earliest of these was used as the date of HIV diagnosis.

### 2.3 Rituximab funding

Rituximab was funded by the New Drug Funding Program (NDFP) in Ontario as of January 2, 2001 for patients with aggressive‐histology lymphoma who have not received previous treatment for aggressive‐histology lymphoma (eg, must be first‐line treatment), and not known to have HIV. As of February 2, 2015, the NDFP began funding rituximab for patients with HIV‐related CD20+ B‐cell lymphoma having CD4 counts >50/mm^3^, again having not received prior treatment for aggressive‐histology lymphoma (eg, must be first‐line treatment). Both policies funded a dose of 375 mg/m^2^ on day 1 of a standard CHOP (or CHOP‐like) regimen for 6‐8 cycles. As this is a real‐world study, we acknowledge that HIV+ patients may have received rituximab under the HIV− policy. However, we do not expect HIV− patients to receive rituximab under the HIV+ policy since HIV‐related information is needed to verify eligibility (eg, CD4 count).

### 2.4 Anti‐neoplastic activity

Administration of rituximab was identified using the NDFP and the Activity Level Reporting database. The NDFP provides public reimbursement for all patients who meet clinical eligibility criteria, as described earlier. We therefore supplemented this using the Activity Level Reporting database, which includes chemotherapy data for all Ontario hospitals, but incomplete coverage for community hospitals prior to 2014. For all other intravenous systemic therapies (eg, CHOP or CHOP‐like), we used the Activity Level Reporting database. Only chemotherapy administered within 6 months after diagnosis was considered relevant as a treatment modality, allowing for 30 days before diagnosis as a buffer.

We classified patients as having received CHOP or a CHOP‐like regimen if they received combinations of cyclophosphamide, vincristine, doxorubicin, etoposide, methotrexate, cytarabine, or ifosfamide (eg, CHOP, EPOCH, CHEOP, CODOXM, CHOMP, CEOP, IVAC). We classified any remaining patients (HIV+ patients only) as having received rituximab alone, no chemotherapy, or another regimen.

### 2.5 Cohorts

**FIGURE 1:**
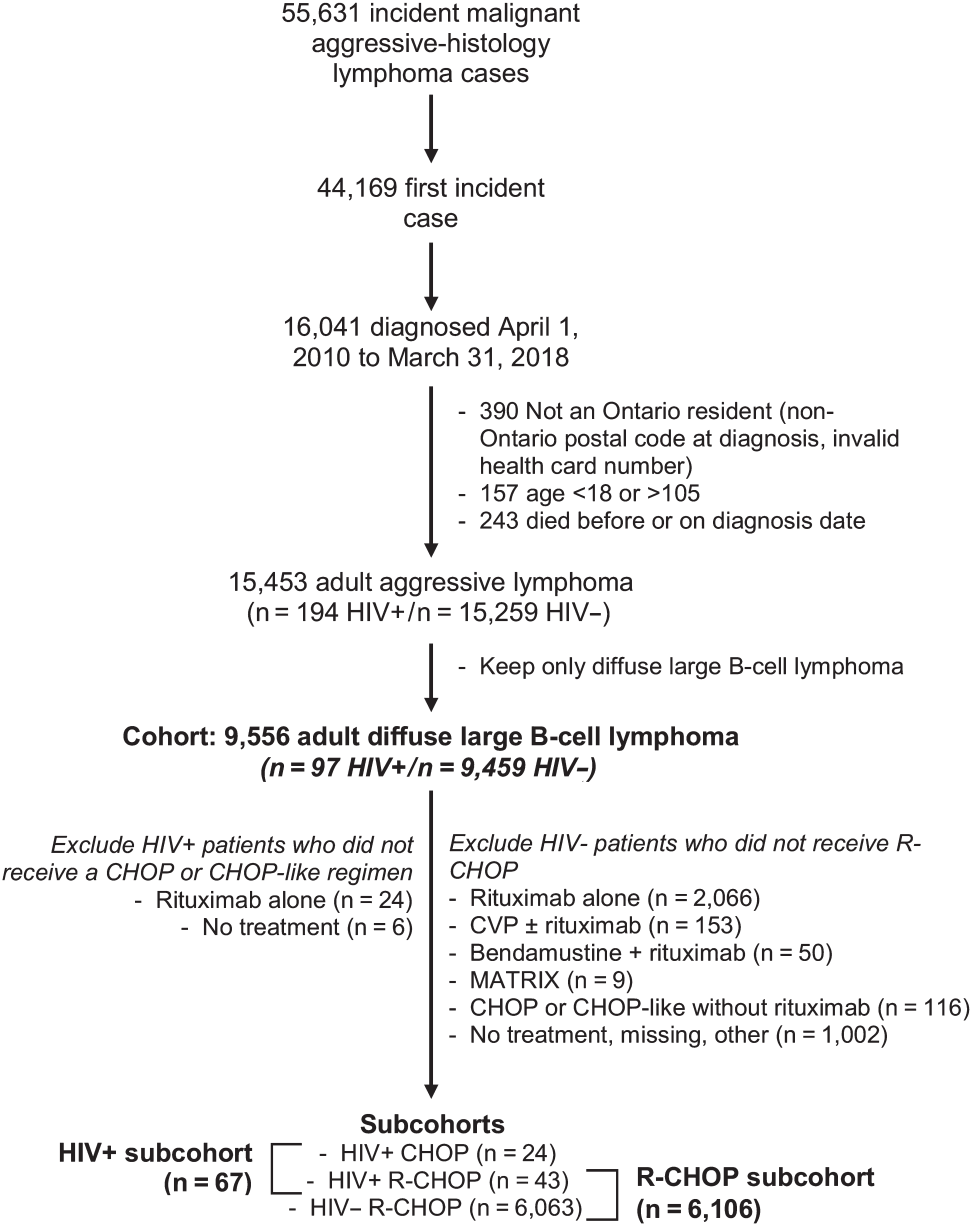
Patient selection. Lymphoma cases were identified from the Ontario Cancer Registry. HIV, human immunodeficiency virus

In the main cohort, we included all adult DLBCL. In the HIV+ subcohort, we only included HIV+ patients who received CHOP or CHOP‐like chemotherapy with or without rituximab (Figure 1). In this cohort of HIV+ DLBCL, we explored the effect of rituximab on clinical outcomes. In a separate second subcohort, we evaluated patients who received R‐CHOP and explored the effect of HIV on clinical outcome. Since R‐CHOP is the standard of care for patients with HIV− lymphoma, patients who received no chemotherapy, CHOP alone, rituximab alone, or any other regimen were excluded as these are likely to be confounded by unknown factors and may overestimate the effect of rituximab.

### 2.6 Outcomes

The primary outcome was overall survival. We censored patients on the date of last follow‐up, which was defined as the most recent date they accessed the healthcare system through the Ontario Health Insurance Program. We obtained death dates from the Ontario Cancer Registry, supplemented with the Registered Persons Database. Secondary outcomes included unscheduled emergency department (ED) visits (National Ambulatory Care Reporting System) and hospitalizations (evidence of a hospital admission in the Discharge Abstract Database) within the first year after treatment.

### 2.7 Covariates

We obtained sociodemographic characteristics from the 2006 Census using the patients' postal code at the time of diagnosis. Among HIV+ patients receiving rituximab through the NDFP, data were available at the time of enrollment (application to receive rituximab) for CD4 count and Eastern Cooperative Oncology Group (ECOG) performance status. To assess comorbidity, we used the Charlson Comorbidity Index using the Discharge Abstract Database and National Ambulatory Care Reporting System looking back 3 years before diagnosis. Central nervous system involvement was inferred using evidence of either brain radiation, brain surgery, a secondary brain cancer diagnosis, or a lymphoma topography involving the central nervous system (Appendix A1).

### 2.8 Statistical methods

We report means (standard deviation, SD), median (25th, 75th percentiles), and N (%), where appropriate. We used logistic regression to report factors associated with dichotomous outcomes (eg, HIV+ vs HIV−, R‐CHOP vs CHOP), reporting odds ratios (OR) with 95% confidence intervals (CI). We used Cox proportional hazards regression for survival analysis, reporting hazard ratios (HR) with 95% CI and presented Kaplan‐Meier plots. For multivariable models, we included all covariates we considered clinically appropriate (no selection mechanism was used). When sample sizes were small, we aggregated levels of some predictors (eg, income quintile, immigrant density, comorbidity) and excluded covariates that had *P* > .2 on univariate analysis. We used Statistical Analysis Software v9.4 (Cary, NC, USA) for all analyses. We obtained ethics approval from Sunnybrook Health Sciences Centre (REB #002‐2019).

## 3 RESULTS

A total 15 453 patients with a first‐ever aggressive‐histology lymphoma were identified after exclusions were applied (Figure 1). Among HIV+ patients, there were <10 cases of T‐cell‐rich large B‐cell lymphoma, mediastinal (thymic) large B‐cell lymphoma, some lymphoma (eg, not otherwise specified), Castleman disease, or Burkitt lymphoma who received rituximab. Thus, in order to evaluate the effectiveness of rituximab in the presence of HIV without confounding by histology, only diffuse large B‐cell lymphomas were included for all subsequent analyses (N = 9556). A total 97/9556 (1.0%) were HIV+, of whom 49 (51%) received rituximab within 6 months after diagnosis. Among HIV− patients, 6063 (63%) received R‐CHOP within 6 months after diagnosis. The time until first treatment was a median 30 (IQR 18, 46) days after diagnosis.

### 3.1 Factors associated with HIV positivity among all DLBCL

Compared with HIV− patients (n = 9459), HIV+ patients (n = 97) were younger (mean 50.9 [SD 12.0] vs 68.1 [SD 14.3] years of age, *P* < .0001), more likely to be male (86% vs 54%, *P* < .0001), resided predominantly in more immigrant‐dense neighborhoods (71% vs 39%, *P* < .0001), and resided in lower‐income neighborhoods (51% vs 37%, *P* = .02) (Table 1).

**TABLE 1:** Factors associated with HIV status among all DLBCL (N = 9556)

	HIV+ (N = 97)^a^	HIV− (N = 9459)	HIV+ vs HIV−	
			Adjusted OR (95% CI)^b^	*P*‐value
Age at diagnosis (y)^c^	50.9 (12.0)	68.1 (14.3)	0.55 (0.49‐0.62)	<.0001
Sex				
Female	14 (14%)	4352 (46%)	1.0 (ref)	<.0001
Male	83 (86%)	5107 (54%)	4.60 (2.59‐8.17)	
Urban residence^d^				
Urban	>93%	8142 (86%)	1.0 (ref)	.19
Rural	<6	1317 (14%)	0.44 (0.13‐1.48)	
Immigrant density^d^				
Least dense	27 (29%)	5764 (61%)	1.0 (ref)	<.0001
Mid‐to‐most dense	67 (71%)	3623 (39%)	2.93 (1.82‐4.73)	
Income quintile^d^				
Highest 3 quintiles	46 (49%)	5957 (63%)	1.0 (ref)	.02
Lowest 2 quintiles	48 (51%)	3451 (37%)	1.67 (1.10‐2.56)	
Charlson Comorbidity Index^e^				
0/missing	73 (75%)	6443 (68%)	1.0 (ref)	.79
1+	24 (25%)	3016 (32%)	1.07 (0.65‐1.75)	
Era^f^				
Before February 2, 2015	58 (60%)	5494 (58%)	1.0 (ref)	.93
After February 2, 2015	39 (40%)	3695 (42%)	0.98 (0.64‐1.50)	
Central nervous system involvement				
No	90 (93%)	8945 (95%)	1.0 (ref)	.64
Yes	7 (7%)	514 (5%)	1.21 (0.55‐2.70)	
Treatment‐related characteristics				
Rituximab				
No	48 (49%)	7169 (75%)	N/A	N/A
Yes	49 (51%)	2290 (24%)		
Regimen				
R‐CHOP	30 (31%)	5442 (58%)	N/A	N/A
CHOP	15 (15%)	116 (1%)		
CEOP ± rituximab	0 (0%)	173 (2%)		
CHOEP/EPOCH ± rituximab	<6	61 (1%)		
CHOMP ± rituximab	12 (12%)	297 (3%)		
CODOXM ± rituximab	<6	90 (1%)		
Rituximab alone	24 (25%)	2066 (22%)		
other or missing	6 (6%)	1214 (13%)		
Among HIV+ patients receiving rituximab (N = 43)				
Median (IQR) time from HIV diagnosis until start of rituximab^g^	22 (2, 136) months	–	–	–
CD4 cell count on enrollment of rituximab	N = 26 133 (86, 334)	–	–	–
Eastern Cooperative Oncology Group				
0	9 (32%)	–	–	–
1‐2	19 (68%)	–	–	–

Abbreviations: HIV, human immunodeficiency virus; IQR, interquartile range (25th, 75th percentile); N/A, comparison not assessed.

^a^ Evidence of at least 3 HIV (human immunodeficiency virus) diagnostic codes in the Ontario Health Insurance Program database within 3 consecutive years.

^b^ Adjusted for age, sex, urban residence, income, immigrant density, comorbidity, era, and central nervous system involvement of the primary disease.

^c^ Odds ratio (OR) and 95% confidence interval (CI) reflect a 10‐year increase in age.

^d^ Source: (or adapted from) Statistics Canada Postal Code Conversion File and Postal Code Conversion File Plus (June 2017) which is based on data licensed from Canada Post Corporation. The patients' postal code at diagnosis was used.

^e^ Excludes cancer and HIV status.

^f^ Rituximab for HIV+ lymphoma was funded in Ontario by the New Drug Funding Program on February 2, 2015.

^g^ Excluding those diagnosed with HIV after diagnosis with lymphoma (n < 6).

### 3.2 Factors associated with receiving rituximab among all DLBCL patients

Patients who received rituximab were a mean 66.1 (SD 13.9) years of age at diagnosis, were mostly male (55%), and resided in a higher‐income neighborhood (23% in the highest) (Appendix A2). After adjusting for sociodemographic and clinical factors, patients were more likely to receive rituximab if they were younger (OR 0.62 (0.60‐0.65) per 10‐years), male (OR 1.19 (1.07‐1.32)), lived in a higher‐income neighborhood (*P* = .04), lived in a less immigrant dense neighborhood (*P* = .001), had fewer comorbidities (*P* < .0001), had no central nervous system involvement (OR 1.9 (8.84‐13.5)), and were HIV− (OR 8.26 (5.26‐13.0)). The use of rituximab was more likely after the NDFP funding change on February 2, 2015 (OR 1.15 (1.04‐1.28)), and this increase was significantly higher (*P*‐interaction = .0004) among HIV+ patients (OR 14.7 (3.56‐61.1), *P* = .0002) than HIV− patients (OR 1.13 (1.01‐1.25), *P* = .03). Conversely, HIV− patients were more likely to receive rituximab in the prefunding period (OR 17.3 (9.39‐32.1)) than the postfunding period (OR 2.44 (1.06‐5.62)).

### 3.3 The prognostic effect of rituximab among HIV+ patients: R‐CHOP vs CHOP

**FIGURE 2:**
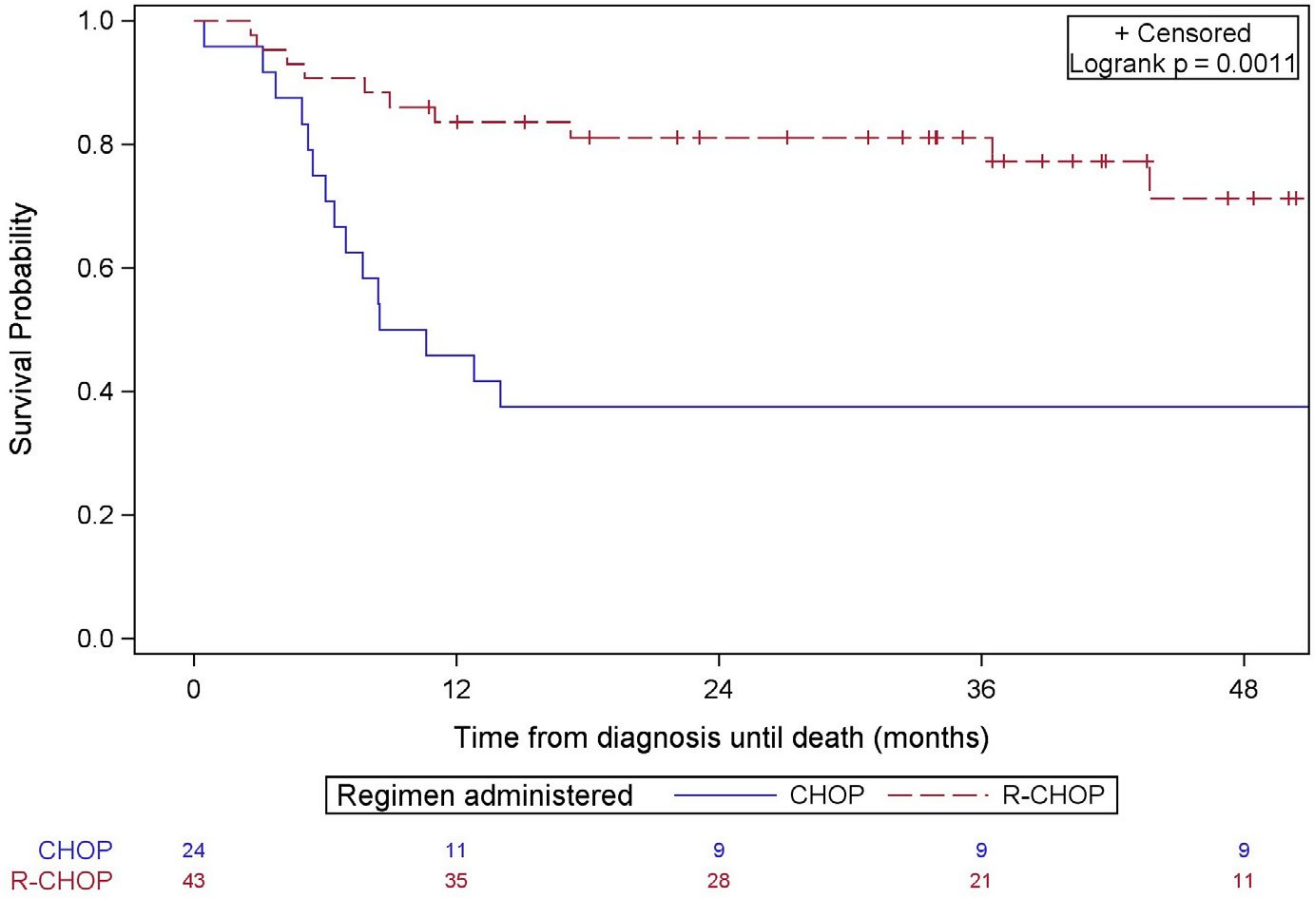
Kaplan‐Meier plot for overall survival by chemotherapy regimen among patients with human immunodeficiency virus (HIV)‐related aggressive lymphoma. Patients received standard chemotherapy (CHOP) or CHOP with rituximab (R‐CHOP)

In the HIV+ subcohort, the standard of care during the study period was CHOP or R‐CHOP, and so we restricted analyses to patients who received either of these regimens (N = 67; Figure 1). Addition of rituximab yielded a fourfold survival advantage compared with standard CHOP (crude HR 0.28 (0.13‐0.63)) (Figure 2). After adjustment for age, neighborhood income quintile, neighborhood immigrant density and comorbidity, the survival benefit of adding rituximab remained unchanged (HR 0.29 (0.13‐0.66)) (Table 2). There were no other significant prognostic factors.

**TABLE 2:** Subcohort analysis: factors associated with overall survival among HIV+ patients receiving CHOP

	HIV+ patients (N = 67)				
	N (%)	Unadjusted HR (95% CI)	*P*‐value	Adjusted HR (95% CI)^a^	*P*‐value
Age at diagnosis, per 10 y	50.8 (SD 11.9)	1.08 (0.78‐1.51)	.64	1.01 (0.71‐1.44)	.96
Sex					
Female	8 (12%)	1.0 (ref)	.66	–	–
Male	59 (88%)	0.79 (0.27‐2.29)			
Urban residence^b^					
Urban	<6	1.0 (ref)	–	–	–
Rural	>90%	N/A			
Income quintile^b^					
Lowest 2 quintiles	34 (53%)	1.0 (ref)	.50	1.0 (ref)	.56
Highest 3 quintiles	30 (47%)	1.31 (0.60‐2.85)		1.10 (0.81‐1.49)	
Immigrant density^b^					
Mid‐to‐most dense	43 (67%)	1.0 (ref)	.66	1.0 (ref)	.61
Least dense	21 (33%)	0.83 (0.37‐1.88)		1.15 (0.67‐1.96)	
Charlson Comorbidity Index^c^					
0/missing	56 (84%)	1.0 (ref)	.03	1.0 (ref)	.07
1+	11 (16%)	2.65 (1.10‐6.40)		1.73 (0.96‐3.11)	
Era^d^					
Before February 2, 2015	37 (55%)	1.0 (ref)	.61	–	–
After February 2, 2015	30 (45%)	0.81 (0.25‐1.85)			
Central nervous system					
Involved	<6	1.0 (ref)	–	–	–
Not involved	>90%	N/A			
Regimen					
CHOP or CHOP‐like	24 (36%)	1.0 (ref)	.002	1.0 (ref)	.004
R‐CHOP or R‐CHOP‐like	43 (64%)	0.28 (0.13‐0.63)		0.29 (0.13‐0.66)	

Abbreviations: CI, confidence interval; HIV, human immunodeficiency virus; HR, hazard ratio.

^a^ Adjusted for age, income quintile, immigrant density, comorbidity, and regimen. Variables excluded from the model have too few patients in a given stratum (eg, <10) or were uninformative (eg, era is strongly correlated to regimen).

^b^ Source: (or adapted from) Statistics Canada Postal Code Conversion File and Postal Code Conversion File Plus (June 2017) which is based on data licensed from Canada Post Corporation. The patients' postal code at diagnosis was used.

^c^ Excluding cancer and HIV.

^d^ Rituximab for HIV+ lymphoma was funded in Ontario by the New Drug Funding Program on February 2, 2015.

### 3.4 **The prognostic effect of HIV among patients receiving R‐CHOP: HIV+ vs HIV**−

In the second subgroup of patients receiving R‐CHOP (N = 6106), the presence of HIV did not affect overall survival (unadjusted HR 0.73 (0.39‐1.36)). After adjustment for clinical and demographic characteristics, overall survival was worse for patients who were older (HR 1.40 (1.34‐1.45) per 10‐year increment), male (HR 1.17 (1.07‐1.28)), lived in a lower income neighborhood (HR 1.35 (1.17‐1.56) for the least vs the highest income quintile), and had more comorbidity (HR 1.67 (1.40‐1.99) for 3+ vs no comorbidity), but the presence of HIV again was not prognostic (HR 1.12 (0.60‐2.10)) (Table 3). In an effort to eliminate the potential effect of selection bias present before the HIV+ funding date of rituximab (eg, healthier HIV+ patients may have been preferentially selected to receive rituximab before February 2015), we conducted a sensitivity analysis restricted to the postfunding era; HIV status remained unassociated with overall survival (HR 1.15 (0.47‐2.79)).

**TABLE 3:** Subcohort analysis: factors associated with overall survival among patients receiving rituximab

	Patients receiving rituximab with a CHOP or CHOP‐like regimen (N = 6106)						
		Unadjusted model		Adjusted		Adjusted, new era only	
	N (%)	HR (95% CI)	*P*‐value	HR (95% CI)^a^	*P*‐value	HR (95% CI)^a^, ^b^	*P*‐value
Age at diagnosis, per 10 y	65.8 (SD 13.7)	1.43 (1.37‐1.48)	<.0001	1.40 (1.34‐1.45)	<.0001	1.34 (1.26‐1.43)	<.0001
Sex							
Female	2742 (45%)	1.0 (ref)	.004	1.0 (ref)	.0006	1.0 (ref)	.71
Male	3364 (55%)	1.14 (1.04‐1.24)		1.17 (1.07‐1.28)		1.03 (0.89‐1.19)	
Urban residence^c^							
Urban	5185 (85%)	1.0 (ref)	.15	1.0 (ref)	.86	1.0 (ref)	.14
Rural	921 (15%)	1.09 (0.97‐1.23)		1.01 (0.89‐1.15)		0.85 (0.69‐1.06)	
Income quintile^c^							
Highest	1387 (23%)	1.0 (ref)	.0004	1.0 (ref)	.0007	1.0 (ref)	.29
Mid‐high	1349 (22%)	1.01 (0.88‐1.16)		1.04 (0.91‐1.19)		0.96 (0.77‐1.20)	
Middle	1217 (20%)	1.15 (1.00‐1.31)		1.14 (0.99‐1.31)		1.05 (0.85‐1.30)	
Mid‐low	1179 (19%)	1.13 (0.98‐1.29)		1.10 (0.96‐1.26)		0.86 (0.68‐1.09)	
Lowest	947 (16%)	1.33 (1.16‐1.53)		1.35 (1.17‐1.56)		1.11 (0.87‐1.41)	
Immigrant density^c^							
Least dense	3920 (65%)	1.0 (ref)	.28	1.0 (ref)	.42	1.0 (ref)	.88
Mid‐dense	1330 (22%)	0.96 (0.86‐1.07)		1.03 (0.92‐1.15)		1.05 (0.87‐1.26)	
Most dense	801 (13%)	0.90 (0.78‐1.03)		0.92 (0.80‐1.07)		1.03 (0.92‐1.30)	
Comorbidity^d^							
Missing	898 (15%)	0.72 (0.62‐0.83)		0.75 (0.65‐0.87)		0.66 (0.51‐0.96)	<.0001
0	3620 (59%)	1.0 (ref)	<.0001	1.0 (ref)	<.0001	1.0 (ref)	<.0001
1	860 (14%)	1.32 (1.17‐1.50)		1.18 (1.04‐1.34)		1.16 (0.95‐1.42)	
2	447 (7%)	1.92 (1.66‐2.23)		1.64 (1.41‐1.90)		1.66 (1.31‐2.10)	
3+	281 (5%)	2.11 (1.77‐2.50)		1.67 (1.40‐1.99)		1.82 (1.37‐2.44)	
Era^e^							
<February 2, 2015	3241 (53%)	1.0 (ref)	.56	1.0 (ref)	.54	–	–
≥February 2, 2015	2865 (47%)	1.03 (0.94‐1.13)		0.97 (0.88‐1.07)			
CNS involvement							
No	6005 (98%)	1.0 (ref)	.20	1.0 (ref)	.17	–	–
Yes	101 (2%)	0.78 (0.53‐1.14)		0.76 (0.52‐1.12)			
HIV							
Negative	6063 (99%)	1.0 (ref)	.32	1.0 (ref)	.72	1.0 (ref)	.76
Positive	43 (1%)	0.73 (0.39‐1.36)		1.12 (0.60‐2.10)		1.15 (0.47‐2.79)	

Abbreviations: CI, confidence interval; HIV, human immunodeficiency virus; HR, hazard ratio.

^a^ Adjusted for age, sex, urban residence, income quintile, immigrant density, comorbidity, era, and HIV status.

^b^ Subgroup analysis restricted to the postfunding era.

^c^ Source: (or adapted from) Statistics Canada Postal Code Conversion File and Postal Code Conversion File Plus (June 2017) which is based on data licensed from Canada Post Corporation. The patients' postal code at diagnosis was used.

^d^ Charlson Comorbidity Index, excluding cancer and HIV.

^e^ Funding of rituximab for HIV+ patients was implemented in Ontario by the New Drug Funding Program on February 2, 2015.

### 3.5 Secondary outcomes: hospitalizations and ED visits

In the subgroup of patients receiving R‐CHOP, 29/43 (67%) HIV+ patients and 3980/6063 (66%) HIV− patients had an ED visit within the first year of treatment (*P* = .80). Similarly, 25/43 (58%) HIV+ patients and 3159 (52%) HIV− patients had a hospital admission within the first year of diagnosis (*P* = .43). We did not compare hospitalizations or ED visits in the HIV+ subgroup since patients who received CHOP had a median survival of only 8.4 months, and any differences in these outcomes would be attributable to longer follow‐up (Figure 2).

## 4 DISCUSSION

Among patients with diffuse large B‐cell lymphoma, rituximab usage increased over time, particularly among HIV+ patients. When used in addition to standard chemotherapy, rituximab significantly and markedly improved overall survival among patients with HIV. Moreover, among patients receiving R‐CHOP, HIV positivity was not associated with worse overall survival and did not result in more patients admitted to hospital or visiting the ED within one year after diagnosis.

We undertook this study because, in the face of limited evidence at the time, Ontario made the decision to fund rituximab specifically for HIV‐related aggressive lymphomas. This funding decision was made acknowledging the promising evolution of clinical evidence and the potential benefit of data collection for the purposes of establishing real‐world safety and effectiveness of the drug. The most substantial evidence at the time was from a phase 3 trial published by the US AIDS Malignancy Consortium (AMC), early in the era of HIV anti‐retroviral treatment.^21^ In this randomized controlled trial, Kaplan et al compared 8‐cycles of R‐CHOP with CHOP in patients with HIV‐related aggressive‐histology B‐cell lymphoma. The experimental arm included an unconventional extended maintenance phase of rituximab, despite evidence suggesting this practice does not offer benefit.^25^, ^26^ Individuals who received rituximab experienced an improved lymphoma‐related survival but this was offset by increased infectious‐related mortality. Many of these deaths occurred in individuals with profound immunodeficiency (CD4 count <50/mm^3^) and during the maintenance phase of rituximab therapy.

The AMC trial was difficult to interpret, given the unusual dosing of rituximab, use of obsolete antiretroviral combinations, and inclusion of patients with severe immunodeficiency (with CD4+ cell counts <50/mm^3^). The current management of HIV‐related lymphoma may have evolved since this earlier study. Modern HAART is not associated with added myelosuppression and an emphasis on compliance has led to immune reconstitution even during the course of chemotherapy. Moreover, antibiotic prophylaxis is routine, maintenance rituximab is not standard practice, and more recent phase II and population‐based studies completed in this era have suggested excellent disease control without the same propensity for causing infectious toxicities suggested by the earlier Kaplan trial.^19^, ^27^, ^28^, ^29^, ^30^, ^31^, ^32^ Our results are consistent with this evolution in literature and practice. In the current era, the benefit of rituximab appears to be safely realized when administered with concomitant immune reconstitution via HAART, thoughtful antibiotic prophylaxis, and careful selection of patients according to CD4+ cell count.

This study has some limitations. First, the prevalence of HIV in this population is low, despite pooling nine years of data. Thus, adjustment for important clinical factors required categorization (particularly in the HIV+ subgroup) or is only available for the HIV+ patients who received rituximab (eg, ECOG, CD4+ T‐cell count—information needed to inform funding eligibility). This may result in residual confounding. Second, receipt of R‐CHOP or CHOP is likely governed by various clinical factors that are unavailable from our administrative datasets, including International Prognostic Index, stage, or prognostic biomarkers or genetic factors. In the HIV+ subgroup, we omitted patients who received no treatment, rituximab only, or any other regimen to reduce the likelihood of confounding by indication. Unlike the HIV− patients where R‐CHOP is the standard of care, the use of R‐CHOP or CHOP among HIV+ patients is less clear and therefore less likely to be subjected to the same degree of confounding (particularly in the era before funding). Given these restrictions, the results of this study are only generalizable to patients who are healthy enough to receive CHOP (patients who receive CHOP are generally healthy enough to also receive rituximab). Despite this, analyses of R‐CHOP vs CHOP in the HIV+ subgroup of patients required data from the prefunding era. During this time, patients who received R‐CHOP may have been the healthiest patients (selection bias). Including or excluding these patients would have the effect of overestimating the survival effect of R‐CHOP in the HIV+ subgroup, and without additional data, this bias cannot be eliminated. Despite this, our estimates are aligned with the literature and clinical expectation. Third, missing data on CHOP before 2014 may have led to some selection bias since these patients were eliminated from certain subgroups. Fourth, we only included patients with DLBCL. Additional studies have demonstrated the effectiveness of rituximab in patients with HIV‐associated Burkitt lymphoma, although such studies have small sample sizes due to the dearth of HIV+ patients with this histology or used historical data as comparators.^33^, ^34^, ^35^ Although our results are generalizable only to DLBCL, there is little reason to suspect a different result for other histologic subtypes. Finally, we acknowledge the possibility for misclassification of HIV status. Despite applying a previously validated algorithm with high sensitivity and specificity, the low prevalence of HIV will result in a low positive predictive value (we estimate 71% assuming a prevalence of 1%).^24^ However, this is a conservative estimate because the prevalence of HIV in a population of aggressive‐histology lymphoma patients is expected to be higher than 1%. Moreover, the strength of the prognostic effect of rituximab in the HIV+ subgroup analysis (Table 2) was high and unlikely to be due entirely to misclassification.

In conclusion, R‐CHOP similarly improved overall survival among HIV+ patients as observed in the general lymphoma population. In the current era, HIV+ patients with reconstituted immunity (CD4 counts >50/mm^3^) appear to tolerate the same therapy generally applied to the non‐HIV population without the need for dose adjustment or omission of anti‐CD20 therapy; there is little evidence demonstrating additional risk on hospitalization or ED visits.

## CONFLICT OF INTEREST

The authors have no conflicts of interest to disclose. Dr Chan is supported by the Canadian Centre for Applied Research in Cancer Control (ARCC). The ARCC receives core funding from the Canadian Cancer Society (grant no. 2015‐703549). Dr Cheung is supported by the Roy and Marjorie Linden Fund and the Joan Fisher and James Rowland Fund.

## AUTHORS' CONTRIBUTIONS

JB, WFD, WI, MC, and KC involved in conceptualization. SH and HG carried out data curation and formal analysis. SH, HG, FB, WFD, WI, MC, and KC involved in methodology and writing of the manuscript.

## DATA SOURCE AND OTHER ACKNOWLEDGMENTS

Parts of this material are based on data and information compiled and provided by CIHI. However, the analyses, conclusions, opinions and statements expressed herein are those of the author, and not necessarily those of Canadian Institute of Health Information.

We acknowledge support of the Ministry of Health and Long Term Care in this report. All views expressed are those of the authors of this report and do not necessarily reflect those of Ontario or the Ministry.

## Supporting information

Supplementary Material

## Data Availability

Research data are not available for sharing due to data sharing restrictions.
