# Supplementary material for: The effectiveness of rituximab and HIV on the survival of Ontario patients with diffuse large B‐cell lymphoma
Source: Cancer Med. 2020 Aug 13;9(19):7072–82. doi: 10.1002/cam4.3362 (PMC7541135; doi:10.1002/cam4.3362)
Supplement: Supplementary file 1 — Supplementary Material [file CAM4-9-7072-s001.docx]

# Supplementary Appendix 1: Definition of central nervous system involvement

Central nervous system involvement was considered present if any of the following were present:

1. brain irradiation 30 days before to 60 days after the diagnosis date using
   1. ALR, radiation to the brain
   2. DAD/NACRS, procedural codes 1AA27, 1AB27, 1AC27, 1AE27, 1AF27, 1AG27, 1AK27, 1AN27, 1AP27, 1AX27, 1AZ27, 1BA27, 1EA27, 1ER27;
2. brain surgery 30 days before to 60 days after the diagnosis date using
   1. DAD/NACRS, procedural codes 1AN87, 1AJ87, 1AA87, 1AC87, 1EA87, 1AP87);
3. diagnosis of a secondary malignant lesion of the brain associated with any hospital procedure 30 days before to 60 days after the diagnosis date using
   1. DAD/NACRS (ICD-10 diagnostic code C798)
4. A topography of the primary lymphoma diagnosis related to the central nervous system, including ICD-10 diagnostic codes C69-C72

# Supplementary Appendix 2: Factors associated with rituximab use (N=9,556)

|  | **Rituximab+**  **(N=7,218)^a^** | **Rituximab-**  **(N=2,338)** | **Rituximab vs. no rituximab** | |
| --- | --- | --- | --- | --- |
|  |  |  | **Adjusted OR (95% CI)^b^** | **p-value** |
| Age at diagnosis (years)^c^ | 66.1 (13.9) | 73.6 (14.5) | 0.62 (0.60-0.65) | <.0001 |
|  |  |  |  |  |
| Sex |  |  |  |  |
| Female | 3,227 (45%) | 1,139 (49%) | 1.0 (ref) | 0.002 |
| Male | 3,991 (55%) | 1,199 (51%) | 1.19 (1.07-1.32) |  |
|  |  |  |  |  |
| Urban residence^d^ |  |  |  |  |
| Urban | 6,179 (86%) | 2,057 (88%) | 1.0 (ref) | 0.08 |
| Rural | 1,039 (14%) | 281 (12%) | 1.16 (0.99-1.36) |  |
|  |  |  |  |  |
| Income quintile^d^ |  |  |  |  |
| Highest | 1,617 (23%) | 427 (18%) | 1.0 (ref) | 0.04 |
| Mid-high | 1,591 (22%) | 499 (22%) | 0.82 (0.70-0.96) |  |
| Middle | 1,416 (20%) | 471 (20%) | 0.86 (0.73-1.02) |  |
| Mid-low | 1,416 (20%) | 469 (20%) | 0.90 (0.76-1.06) |  |
| Lowest | 1,148 (16%) | 466 (20%) | 0.72 (0.61-0.85) |  |
|  |  |  |  |  |
| Immigrant density^d^ |  |  |  |  |
| Least dense | 4,477 (63%) | 1,314 (57%) | 1.0 (ref) | 0.001 |
| Mid-dense | 1,631 (23%) | 588 (25%) | 0.85 (0.75-0.96) |  |
| Most dense | 1,050 (15%) | 421 (18%) | 0.79 (0.68-0.92) |  |
|  |  |  |  |  |
| Charlson Comorbidity Index^e^ |  |  |  |  |
| Missing | 1,003 (14%) | 177 (8%) | 1.22 (1.01-1.46) |  |
| 0 | 4,255 (68%) | 1,081 (46%) | 1.0 (ref) | <.0001 |
| 1 | 1,051 (15%) | 488 (21%) | 0.63 (0.55-0.72) |  |
| 2 | 542 (8%) | 278 (12%) | 0.64 (0.54-0.76) |  |
| 3+ | 367 (5%) | 314 (13%) | 0.36 (0.30-0.43) |  |
|  |  |  |  |  |
| Era^f^ |  |  |  |  |
| Before February 2, 2015 | 4,162 (58%) | 1,390 (59%) | 1.0 (ref) | 0.04 |
| After February 2, 2015 | 3,056 (42%) | 948 (41%) | 1.15 (1.04-1.28) |  |
|  |  |  |  |  |
| Central nervous system |  |  |  |  |
| Involved | 162 (2%) | 359 (15%) | 1.0 (ref) | 0.04 |
| Not involved | 7,056 (98%) | 1,979 (85%) | 10.9 (8.84-13.5) |  |
|  |  |  |  |  |
| HIV status |  |  |  |  |
| Positive | 49 (1%) | 48 (2%) | 1.0 (ref) | <.0001 |
| Negative | 7,169 (99%) | 2,290 (98%) | 8.26 (5.26-13.0) |  |
| ^a^ receipt of rituximab within 6 months after diagnosis  ^b^ adjusted for age, sex, urban residence, income quintile, immigrant density, comorbidity, era, and HIV status  ^c^ odds ratio (OR) and 95% confidence interval (CI) reflect a 10-year increase in age  ^d^ source: (or adapted from) Statistics Canada Postal Code Conversion File and Postal Code Conversion File Plus (June 2017) which is based on data licensed from Canada Post Corporation. The patients’ postal code at diagnosis was used.  ^e^ excludes cancer and HIV (human immunodeficiency virus) status  ^f^ rituximab for HIV+ lymphoma was funded in Ontario by the New Drug Funding Program on February 2, 2015 | | | | |
